# 2394. Comparative Analysis of Behavior Change and Vaccine Uptake Beliefs

**DOI:** 10.1093/ofid/ofad500.2014

**Published:** 2023-11-27

**Authors:** Tyler Walsh, Monique A Lavalas Bright, Jingxia Liu, Jason Newland

**Affiliations:** Washington University School of Medicine in Saint Louis, St. Louis, Missouri; Washington University in St. Louis School of Medicine, St. Louis, Missouri; WUSM, Saint Louis, Missouri; Washington University School of Medicine, Saint Louis, MO

## Abstract

**Background:**

COVID-19 vaccination rates have been wide-ranging among age groups, specifically among the pediatric population. Children under the age of 4 currently representing 12% with at least one dose, while children aged 5-11 represent 39% with at least one dose. We hypothesize that understanding and teaching children and caregivers will cause an increase in capability, opportunity and motivation (COM-B Model) towards the COVID-19 vaccine.

**Figure:**
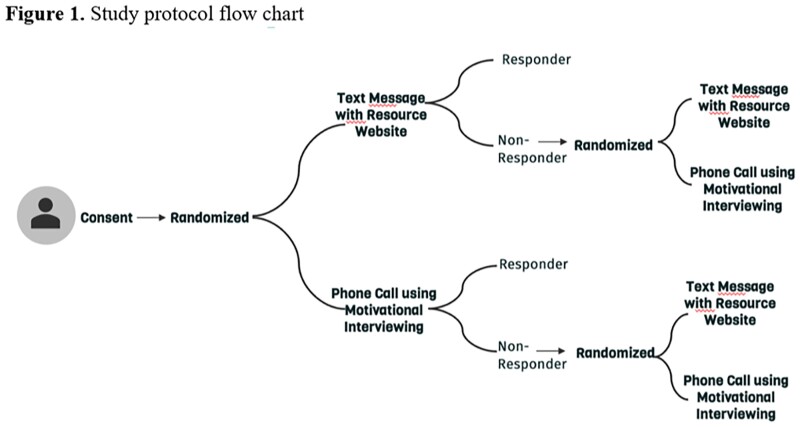

**Methods:**

The study was conducted within St. Louis City and County School Districts. Participants were randomized to one of two interventions, re-randomized after 2 weeks if the participant was not vaccinated, and received follow up assessment surveys. The interventions entail the participant receiving a text message containing information and resources on vaccination or a phone call with a study team member, to further discuss beliefs around the vaccine. The primary outcome is behavioral measures toward vaccine, defined as the average of capability, opportunity and motivation scores. It was collected at three time points: baseline, first follow up and second follow up. A mixed regression model with autoregressive correlation structure, was used to assess the intervention’s effect on the primary outcome.

**Figure:**
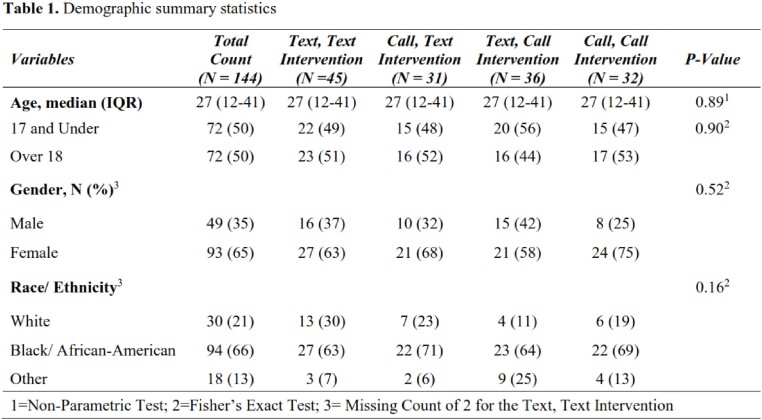

**Figure:**
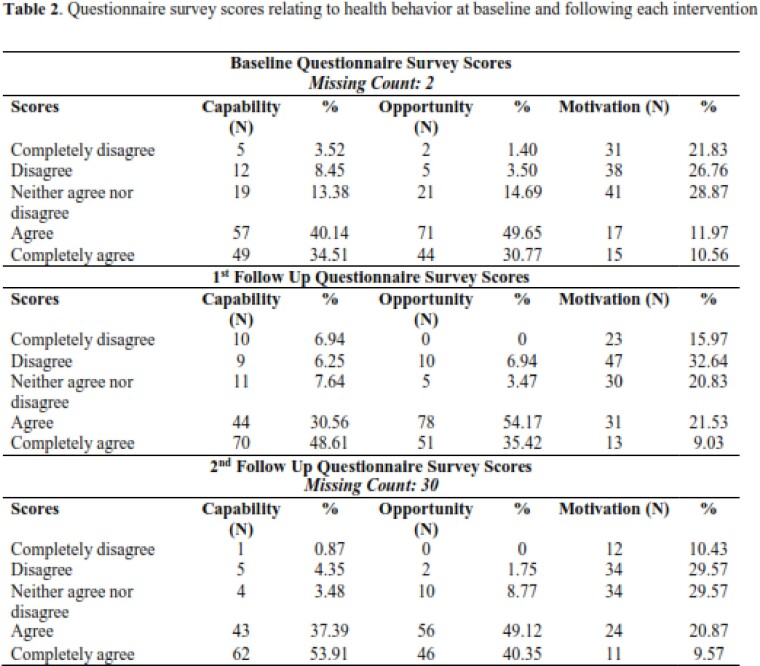

Questionnaire survey scores relating to health behavior at baseline and following each intervention

**Results:**

One hundred and forty-four (144) participants met the eligibility criteria and received both randomizations for this study. Participant agreement of capability (54%) and opportunity (49%) toward the COVID-19 vaccine improved, while motivation (30%) remained disagreed or neither agree nor disagree toward the vaccine. The mixed regression model showed the group intervention was a significant factor across the time points, p-value < 0.001. Specific improvement in LSM for the Call, Text intervention group with significant effect on primary outcome across the time points [baseline LSM: 3.133, (95% CI: 2.865, 3.402); and first follow up LSM: 3.3222 (95% CI: 3.0671, 3.5774)]

**Figure:**
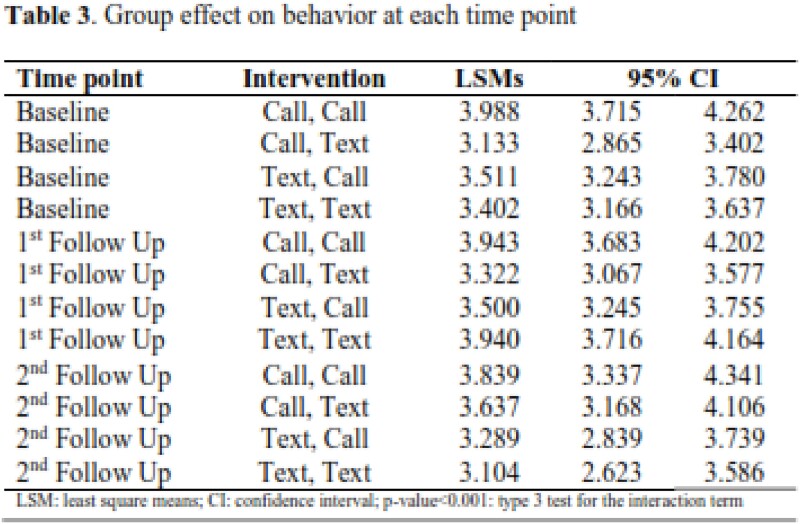

**Figure:**
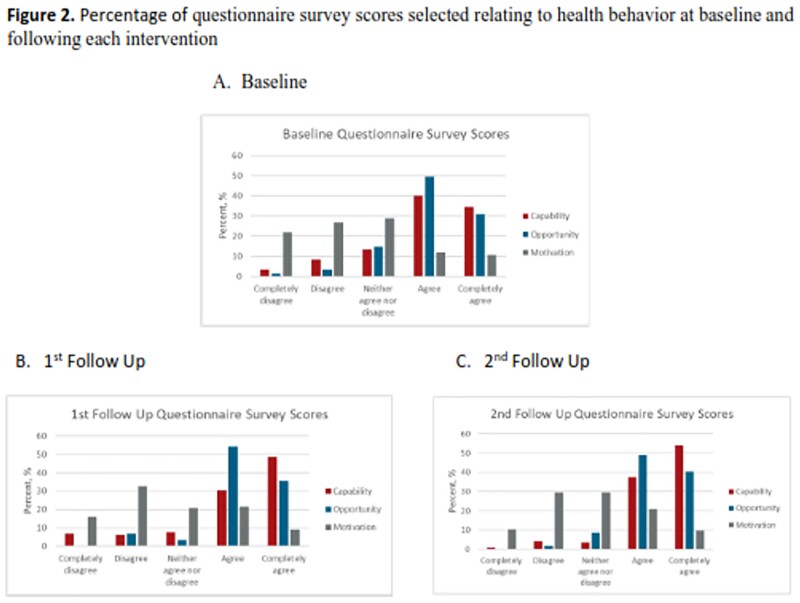

Percentage of questionnaire survey scores selected relating to health behavior at baseline and following each intervention

**Conclusion:**

Participant survey scores improved the most in capability and opportunity of behavior change towards the vaccine, while motivation did not have an improvement. The use of mixed intervention, Call, Text, leads to the most effective behavior towards the vaccine.

**Disclosures:**

**Jason Newland, MD**, Moderna: Grant/Research Support|Pfizer: Grant/Research Support

